# Complication-specific microbial signatures in gestational diabetes mellitus, hypertensive disorders of pregnancy, and fetal macrosomia: a multi-site analysis of the maternal saliva–amniotic fluid–neonatal meconium continuum

**DOI:** 10.3389/fnut.2026.1961007

**Published:** 2026-10-21

**Authors:** Yan Li, Binglong Qiao, Chunling Ma, Can Li, Qingcheng Meng, Minhui Cheng, Chao Li

**Affiliations:** 1 Department of Obstetrics, The Affiliated Hospital of Qingdao University, Qingdao, Shandong, China; 2 Department of Radiology, The Affiliated Hospital of Qingdao University, Qingdao, Shandong, China

**Keywords:** 16S rRNA sequencing, candidate microbial signatures, FM, GDM, HDP, maternal-infant microbial concordance

## Abstract

**Background:**

Gestational diabetes mellitus (GDM), hypertensive disorders of pregnancy (HDP), and fetal macrosomia (FM) are common pregnancy complications threatening maternal–infant health. However, due to a lack of systematic analysis, whether these complications are associated with distinct cross-site microbial patterns along the saliva–amniotic fluid–meconium axis remains unclear.

**Methods:**

In this study, 162 samples (54 each of maternal saliva, amniotic fluid, and neonatal meconium) from 54 mother-neonate pairs (20 normal controls (NC), 18 GDM, 6 FM, and 10 HDP) were analyzed by 16S rRNA gene sequencing.

**Results:**

In the NC group, the microbial composition of meconium was significantly distinct from that of saliva and amniotic fluid, while saliva and amniotic fluid exhibited relatively similar structures. Microbial richness in meconium was significantly reduced in the HDP and FM groups. The HDP group was enriched with *Klebsiella*, *Enterobacter*, and *Sphingomonas*. The FM group was enriched with *Staphylococcus* and *Methyloversatilis*, and the GDM group was enriched with *Bergeyella* and *Helicobacter*. In amniotic fluid samples, the HDP group was enriched with *Emticicia*, the FM group with *Comamonadaceae*, and the GDM group with *Lactobacillus iners*. Cross-site concordance analysis revealed that *Prevotella*, *Neisseria*, *Haemophilus*, and *Streptococcus* exhibited a continuous abundance gradient across saliva, amniotic fluid, and meconium in the HDP group, suggesting potential cross-site microbial transfer. In the FM group, Fusobacterium showed a pattern of detection in amniotic fluid with increased abundance in meconium, while Paucibacter was predominantly detected in meconium, a pattern potentially consistent with maternal contact during delivery. Correlation analyses showed that *Rothia* was positively correlated with triglycerides, *Neisseria* with glycated hemoglobin, and *Methyloversatilis* with pre-pregnancy body mass index (BMI) (*p* < 0.05).

**Conclusion:**

This study is the first to systematically compare the effects of GDM, HDP, and FM on the microbiota of maternal saliva, amniotic fluid, and neonatal meconium, revealing complication-specific microbial signatures and dynamic cross-site concordance patterns along the saliva–amniotic fluid–meconium axis.

## Introduction

GDM, HDP, and FM are the most common complications during pregnancy, seriously threatening maternal and infant health. GDM refers to glucose intolerance first recognized during pregnancy, with a global prevalence of approximately 14.0%, and a history of GDM significantly increases the risk of developing type 2 diabetes postpartum (1, 2). HDP represent a group of conditions characterized by the coexistence of hypertension and pregnancy, and constitute one of the leading causes of maternal and perinatal mortality worldwide (3). FM occurs more frequently in overweight and obese pregnant women and increases the risk of adverse outcomes such as cesarean section, shoulder dystocia, and neonatal birth injuries (4). Although the pathological mechanisms of these complications remain incompletely understood, recent studies suggest that microbial dysbiosis is associated with their development and progression, indicating a link between gut microbiota and these diseases (5–7).

The human microbiome plays a critical role in maintaining metabolic and immune homeostasis during pregnancy (8). Studies have shown that pregnant women with GDM exhibit significant alterations in the gut, oral, and vaginal microbiota, and such dysbiosis can affect early microbial colonization in neonates (9). Crusell et al. found that the gut microbial composition of GDM women differed significantly from that of non-GDM women, and these differences were closely associated with oral glucose tolerance test results (10). In the context of HDP, imbalances in the maternal gut, oral, and placental microbiota are thought to participate in disease pathogenesis through inflammatory responses (11). Furthermore, studies on FM have revealed significant alterations in the microbial composition of the maternal gut and amniotic fluid, with differential taxa associated with neonatal birth weight and fetal ultrasound parameters (12, 13). These lines of evidence suggest that pregnancy complications may be accompanied by site-specific microbial dysbiosis and may be associated with offspring health, potentially involving maternal–infant microbial concordance.

Notably, the pathogenic or protective effects of the microbiota exhibit significant site specificity (14). Maternal oral microbiota can reflect systemic metabolic and inflammatory status, amniotic fluid microbiota directly represents the intrauterine exposure environment of the fetus, and neonatal meconium microbiota reflects the initial colonization of the gut at birth. Existing studies have largely focused on microbial alterations at a single site, lacking systematic analysis of multi-site microbial cross-site concordance (15). Whether the saliva–amniotic fluid–meconium axis reflects maternal-fetal microbial concordance, and whether different pregnancy complications affect this cross-site pattern, remain to be elucidated.

This study represents the first application of 16S rRNA gene sequencing technology to systematically integrate and analyze maternal saliva, amniotic fluid, and neonatal meconium samples, aiming to investigate the effects of GDM, HDP, and FM on maternal–infant microbial composition and cross-site microbial concordance. By comparing the microbial characteristics of different complications across various sample sites, identifying candidate microbial signatures, and exploring their correlations with clinical indicators, this study seeks to characterize site-specific patterns of pregnancy complication-related microbial dysbiosis and the features of potential cross-site microbial transfer. The findings are expected to provide novel microbial targets for early warning and risk assessment of pregnancy-related diseases.

## Materials and methods

### Patient recruitment

This study was conducted at the Affiliated Hospital of Qingdao University and aimed to recruit pregnant women and their newborns. The study protocol was approved by the Ethics Committee of the Affiliated Hospital of Qingdao University (no. QYFYWZLL42371), and all participants (including mothers and guardians of the newborns) signed written informed consent, acknowledging the purpose and potential risks of the study. This study primarily enrolled singleton pregnant women between 20 and 40 years of age, confirmed by ultrasound examination. The diagnosis of GDM followed the latest World Health Organization guidelines: a 75 g oral glucose tolerance test (OGTT) was performed between 24 and 28 weeks of gestation, and a diagnosis was made if at least one of the following criteria was met: fasting blood glucose ≥ 5.1 mmol/L, 1-h blood glucose ≥ 10.0 mmol/L, or 2-h blood glucose ≥ 8.5 mmol/L. The diagnosis of HDP was based on the relevant clinical guidelines. The diagnostic criterion for FM was neonatal birth weight ≥ 4,000 g. Participants diagnosed with more than one of GDM, HDP, or FM were excluded to ensure that the four groups were mutually exclusive. Maternal and infant characteristics were extracted from medical records, including maternal age, parity, pre-pregnancy weight, height, pre-pregnancy BMI, mode of delivery, and antibiotic use during pregnancy. All enrolled participants underwent cesarean delivery and received cefazolin for infection prophylaxis. A total of 162 samples were collected in this study, including 54 each of meconium, amniotic fluid, and maternal saliva. The distribution of each sample type across groups was as follows: NC group (*n* = 20), GDM group (*n* = 18), FM group (*n* = 6), and HDP group (*n* = 10). Baseline clinical characteristics of the four groups are summarized in Supplementary Table S1.

### Sample collection

Maternal saliva, amniotic fluid, and neonatal meconium samples were all collected at the designated hospital of this study. Maternal saliva samples were collected from the floor of the mouth within 24 h before delivery using sterile swabs, immediately placed on dry ice, and subsequently transferred to an −80 °C freezer for storage. Amniotic fluid samples were collected exclusively from patients undergoing elective cesarean section before the onset of labor, with no evidence of premature rupture of membranes or intrauterine infection. Samples were aspirated using a sterile syringe prior to membrane rupture during cesarean delivery, immediately placed into sterile centrifuge tubes, centrifuged at 1,000 × g for 10 min at 4 °C, and the supernatant was aliquoted into sterile cryotubes, rapidly placed on dry ice, and then transferred to −80 °C for storage. The first neonatal meconium after birth was collected as soon as it was passed and confirmed to have typical characteristics (dark, sticky, tar-like). Meconium samples collected at the hospital were scraped from the central part of the diaper (avoiding contamination with urine) using a sterile spoon, placed into sterile cryotubes, immediately placed on dry ice, and subsequently transferred to −80 °C for storage.

### Sample processing and 16S rRNA gene sequencing

Total genomic DNA was extracted from meconium, saliva, and amniotic fluid samples using the TGuide S96 Magnetic DNA Kit (Tiangen Biotech (Beijing) Co., Ltd.) according to the manufacturer’s instructions. The quality and quantity of the extracted DNA were examined by electrophoresis on a 1.8% agarose gel, and DNA concentration and purity were determined using a NanoDrop 2000 UV–Vis spectrophotometer (Thermo Scientific, Wilmington, United States). The hypervariable region V3-V4 of the bacterial 16S rRNA gene was amplified with primer pairs 338F (5’-ACTCCTACGGGAGGCAGCA-3′) and 806R (5’-GGACTACHVGGGTWTCTAAT-3′). Both the forward and reverse 16S primers were tailed with sample-specific Illumina index sequences to allow for deep sequencing. The PCR was performed in a total reaction volume of 20 μL: DNA template 5–50 ng, forward primer (10 μM) 0.3 μL, reverse primer (10 μM) 0.3 μL, KOD FX Neo Buffer 5 μL, dNTP (2 mM each) 2 μL, KOD FX Neo 0.2 μL, and finally ddH₂O up to 20 μL. The amplification conditions were as follows: initial denaturation at 95 °C for 5 min, followed by 20 cycles of denaturation at 95 °C for 30 s, annealing at 50 °C for 30 s, and extension at 72 °C for 40 s, with a final extension at 72 °C for 7 min. The amplified products were purified using an Omega DNA purification kit (Omega Inc., Norcross, GA, United States) and quantified using Qsep-400 (BiOptic, Inc., New Taipei City, Taiwan, ROC). The amplicon library was paired-end sequenced (2 × 250 bp) on an Illumina NovaSeq 6,000 platform.

### Bioinformatic analysis

Qualified sequences with more than 97% similarity thresholds were clustered into operational taxonomic units (OTUs) using USEARCH (version 10.0). Taxonomy annotation of the OTUs was performed based on the Naive Bayes classifier in QIIME2 (16) using the SILVA database (release 138.1) (17) with a confidence threshold of 70%. Alpha diversity indices (ACE, Chao1, and Shannon) were calculated using QIIME2 software to assess the species complexity of each sample, and differences between groups were compared using Student’s t-test. Beta diversity was evaluated by principal coordinate analysis (PCoA) based on Bray-Curtis distances to compare species composition among samples. Linear discriminant analysis (LDA) coupled with effect size (LEfSe) was applied to identify differentially abundant taxa (LDA threshold > 4.0). The impact of clinical indicators on the microbiota was evaluated using PERMANOVA (adonis function, vegan package version 2.6.4), with multiple comparisons corrected using the Bonferroni-Holm method. Correlation analyses between differential microbial taxa and clinical indicators were performed using Pearson correlation coefficients. *p* values were adjusted for multiple testing using the Benjamini-Hochberg false discovery rate (FDR) method, and adjusted *p* < 0.05 was considered statistically significant. The online platform BMKCloud^1^ was used to analyze the sequencing data.

## Results

### Multi-site microbiome characteristics and community differences in the normal control group

We first characterized the microbial composition of saliva, amniotic fluid, and meconium in the NC group to describe the baseline microbial features of these three sites. Alpha diversity analysis revealed significant differences in microbial alpha diversity among meconium, saliva, and amniotic fluid (Figure 1A), with meconium exhibiting significantly richer microbial composition compared to saliva and amniotic fluid. To compare the differences in microbial community structure among the three sample types, PCoA based on Bray-Curtis distances was performed. The results showed that meconium samples were clearly separated from saliva and amniotic fluid samples, while saliva and amniotic fluid exhibited partial overlap (Figure 1B). Venn diagram analysis revealed 65 shared microbial taxa among the three groups, with meconium samples harboring the highest number of unique microbial taxa, suggesting a unique community structure of the neonatal gut microbiota (Figure 1C). Phylum-level microbial composition analysis showed that meconium samples were dominated by Firmicutes and Proteobacteria, while saliva and amniotic fluid samples were rich in Actinobacteria, Bacteroidota, and Firmicutes, with relatively similar compositional structures between the latter two (Figures 1D,E). These results indicate that the microbial composition of meconium is significantly distinct from that of saliva and amniotic fluid, while the microbial community structures of saliva and amniotic fluid are relatively close, suggesting that the neonatal gut microbiota has unique community characteristics and that there may be some microbial similarity between the maternal oral cavity and the intrauterine environment. Further analysis of microbial composition differences among the three sample types at the genus level revealed that *Fusobacterium*, *Haemophilus*, *Prevotella_7*, and *Solobacterium* had significantly higher relative abundances in saliva samples compared to amniotic fluid and meconium samples (*p* < 0.05) (Figures 1F,G).

**Figure 1:**
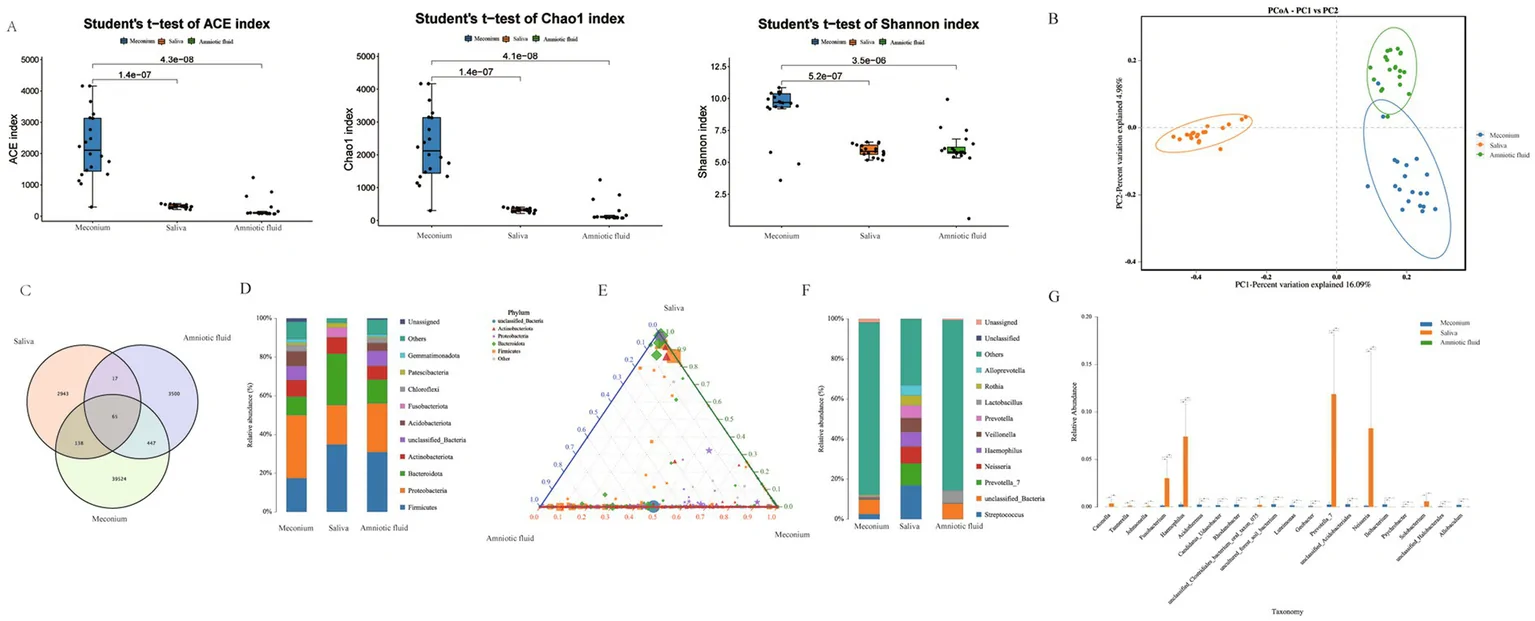
Comparison of microbial composition among saliva, amniotic fluid, and meconium in the NC group. **(A)** Alpha diversity analysis of meconium, saliva, and amniotic fluid samples. **(B)** PCoA based on Bray-Curtis distances, showing clear separation of meconium samples from saliva and amniotic fluid samples, with partial overlap between saliva and amniotic fluid. **(C)** Venn diagram of OTUs among meconium, saliva, and amniotic fluid samples. **(D)** Bar plot showing the relative abundance of microbial taxa at the phylum level among the three groups. **(E)** Ternary plot at the phylum level among the three groups. Each vertex represents one group, and each edge quantifies the species abundance of the correspondingly colored group. Circles represent phylum-level taxa, with circle size indicating the mean relative abundance. **(F)** Bar plot showing the relative abundance of microbial taxa at the genus level among the three groups. **(G)** Bar plot showing significantly different microbial genera among the three groups.

### Comparison of meconium microbiome signatures across pregnancy complication groups

To investigate the impact of pregnancy complications on the neonatal gut microbiota, we compared the meconium microbial composition among the GDM group, FM group, HDP group, and NC group, aiming to identify potential characteristic taxa associated with each complication. The results showed that compared with the NC group, microbial richness in meconium was significantly reduced in the HDP and FM groups (*p* < 0.05), while the GDM group showed a decreasing trend that did not reach statistical significance (Figures 2A–C-left). Genus-level composition analysis revealed that the microbial community structures of meconium samples in the HDP group and NC group were similar, both dominated by *Actinobacteriota*, *Bacteroidota*, *Firmicutes*, and *Proteobacteria*, with only slight differences in the relative abundances of a few low-abundance phyla (Figure 2A-middle). To further identify HDP-associated microbes in neonatal meconium, differential analysis at the genus level was performed between the two groups. The results showed that the abundances of *Klebsiella*, *Enterobacter*, and *Sphingomonas* were significantly higher in the HDP group compared to the NC group (*p* < 0.001) (Figure 2A-right). The microbial composition of meconium samples in the FM group and NC group was dominated by *Streptococcus*, *Bacteroides*, *Klebsiella*, *Sedimenticola*, *Pseudomonas*, and *Staphylococcus*, with higher relative abundances of *Staphylococcus* and *Methyloversatilis* in the FM group (Figure 2B-middle). Differential analysis revealed that *Prevotellaceae* was significantly increased in the FM group (*p* < 0.05) (Figure 2B-right). In the GDM group, the meconium microbial composition showed specific alterations. The relative abundances of *Bifidobacterium* and *Staphylococcus* were higher in the GDM group compared to the NC group, while the relative abundance of Pseudomonas was lower (Figure 2C-right). Differential analysis between the two groups revealed that GDM-associated microbes, including *Bergeyella*, *Helicobacter*, *Mycobacterium*, and *Streptomyces*, were significantly increased in the meconium of the GDM group compared to the NC group (*p* < 0.05) (Figure 2C-right).

**Figure 2:**
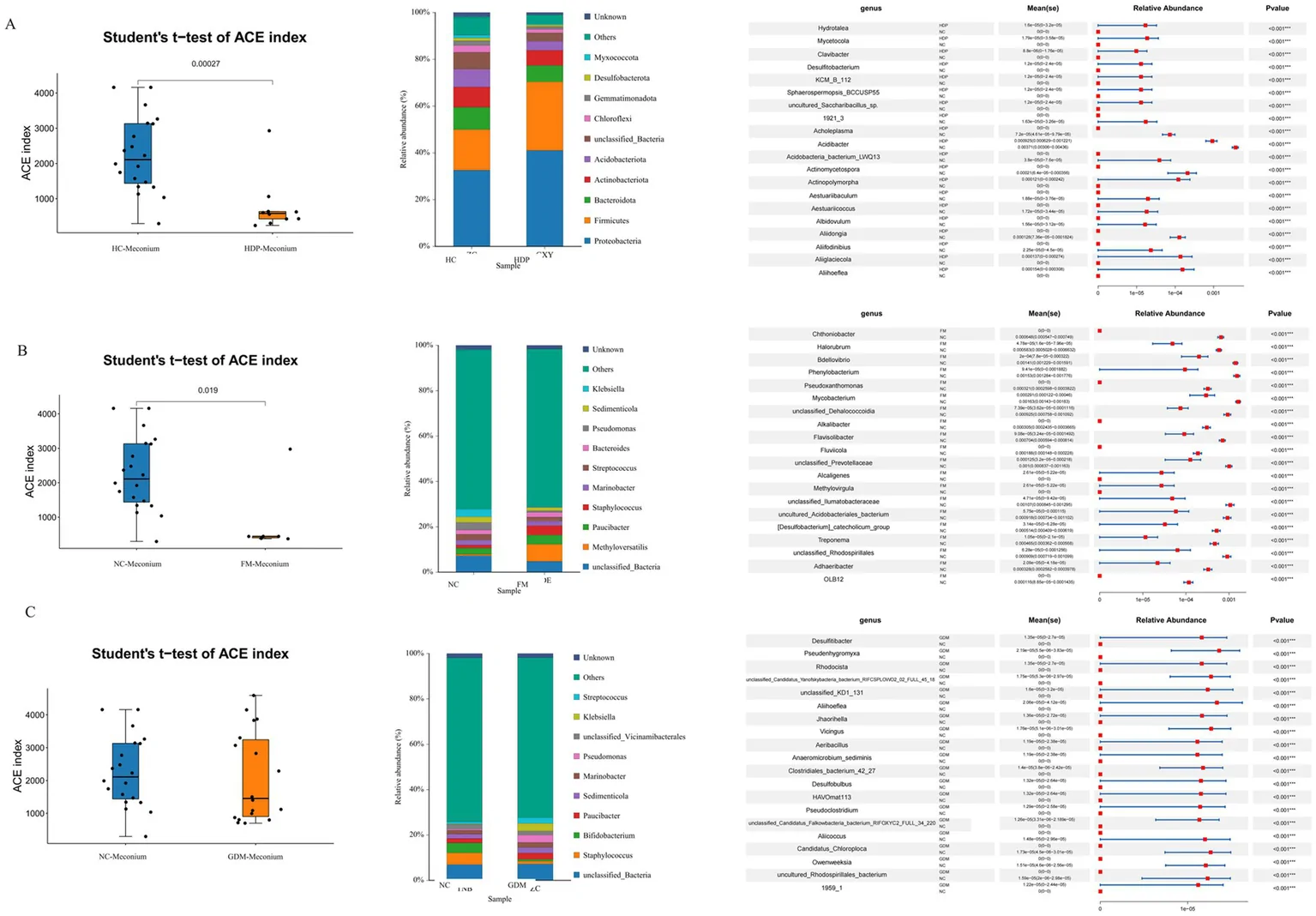
Analysis of meconium microbiome characteristics in newborns associated with hypertensive disorders of pregnancy, gestational diabetes mellitus, and fetal macrosomia. **(A)** HDP group versus NC group: alpha diversity analysis of meconium samples (left); bar plot of phylum-level relative abundance (middle); differential abundance analysis of genus-level microbial taxa between the two groups (right). **(B)** FM group versus NC group: Alpha diversity analysis of meconium samples (left); bar plot of phylum-level relative abundance (middle); differential abundance analysis of genus-level microbial taxa between the two groups (right). **(C)** GDM group versus NC group: Alpha diversity analysis of meconium samples (left); bar plot of phylum-level relative abundance (middle); differential abundance analysis of genus-level microbial taxa between the two groups (right).

### Amniotic fluid microbial signatures in GDM, HDP, and fetal macrosomia

The composition of the amniotic fluid microbiome is closely associated with fetal intrauterine development. To determine whether different pregnancy complications are accompanied by alterations in the intrauterine microenvironment, we analyzed the microbial community structure and differential taxa in amniotic fluid samples across groups. At the genus level, the core microbiota in the HDP group and NC group included *Lactobacillus* and *Staphylococcus*, among which *Emticicia* was significantly higher in the HDP group compared to the control group (*p* < 0.05) (Figure 3A). Composition analysis showed that both the FM group and the NC group were dominated by *Firmicutes*, *Proteobacteria*, and *Bacteroidota* (Figure 3B). At the genus level, Staphyl*o*coccus and *Lactobacillus* exhibited relatively high abundances. Differential analysis revealed that *Erysipelatoclostridiaceae* was significantly increased in the FM group (*p* < 0.05) (Figure 3C). LEfSe analysis further identified *Comamonadaceae* as a differential taxon in the FM group (LDA > 4.0) (Figure 3D). Furthermore, analysis of microbial composition and differential taxa in the GDM group showed that the core microbiota consisted of *Lactobacillus*, *Limosilactobacillus*, and *Ureaplasma*. LEfSe analysis revealed that *Acinetobacter* and *Lactobacillus iners* were significantly enriched in the GDM group (LDA > 4.0) (Figure 3E). These results indicate that different pregnancy complications are accompanied by specific alterations in the amniotic fluid microbiome, suggesting that intrauterine microbial dysbiosis may participate in the pathological processes of these diseases.

**Figure 3:**
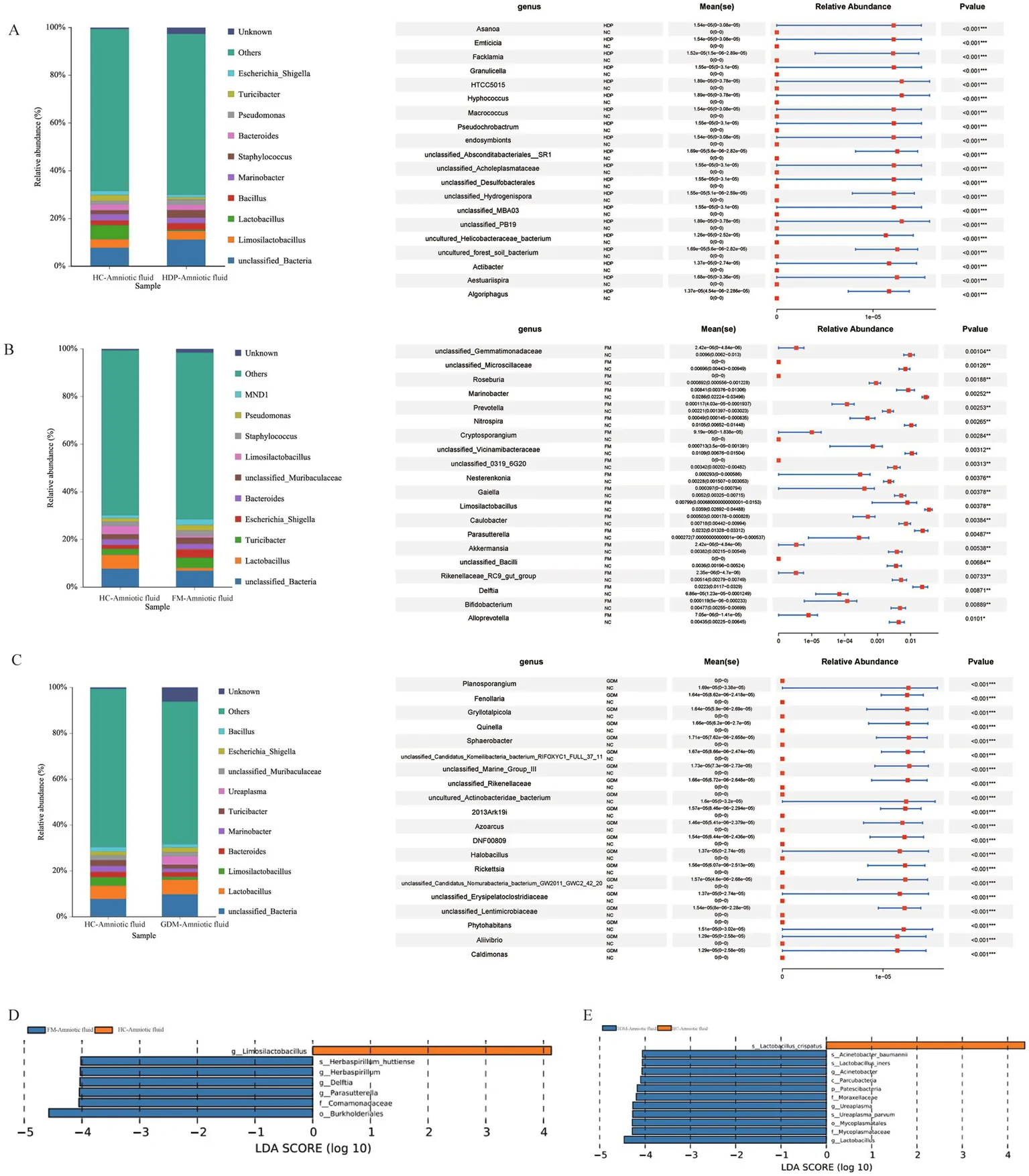
Differential analysis of amniotic fluid microbiome characteristics between different pregnancy complication groups and NC. **(A)** Bar plots of genus-level microbial abundance and significant difference analysis between the HDP group and the NC group. **(B)** Bar plots of genus-level microbial abundance and significant difference analysis between the FM group and the NC group. **(C)** Bar plots of genus-level microbial abundance and significant difference analysis between the GDM group and the NC group. **(D)** LDA score distribution bar plot from LEfSe analysis between the FM group and the NC group (LDA > 4). **(E)** LDA score distribution bar plot from LEfSe analysis between the GDM group and the NC group (LDA > 4).

### Complication-specific alterations of the maternal salivary microbiome

The aforementioned results revealed the impact of different complications on the neonatal gut microbiota and intrauterine environment. Since saliva microbiota can reflect the maternal oral microecological status, to further investigate the relationship between GDM, FM, HDP and the maternal oral microbiota, we analyzed the microbial community structure and differential taxa in saliva samples across groups. The microbial composition of saliva samples in the GDM, FM, and HDP groups was similar to that of the NC group, all dominated by *Veillonella*, *Neisseria*, *Prevotella_7, Haemophilus*, *Alloprevotella*, and *Streptococcus*. Differential analysis showed that *Desulfomicrobium*, *Novosphingobium*, and *Lachnospiraceae* were significantly increased in the HDP group (*p* < 0.05) (Figure 4A). In the FM group, *Streptococcus*, *Rothia*, and *Parvimonas* were significantly increased (*p* < 0.05) (Figure 4B), while in the GDM group, *Lactococcus* and *Arthrobacter* were significantly increased (*p* < 0.05) (Figure 4C). Furthermore, LEfSe analysis revealed that *Neisseria* and *Neisseria sicca* were significantly more abundant in the saliva of HDP patients compared to NCs (Figure 4D). *Alloprevotella_ravazai* was significantly increased in saliva samples of the FM group (Figure 4E). These results indicate that different pregnancy complications are accompanied by specific alterations in the maternal oral microbiota, suggesting that oral microecological dysbiosis may serve as a potential window reflecting metabolic and inflammatory status during pregnancy.

**Figure 4:**
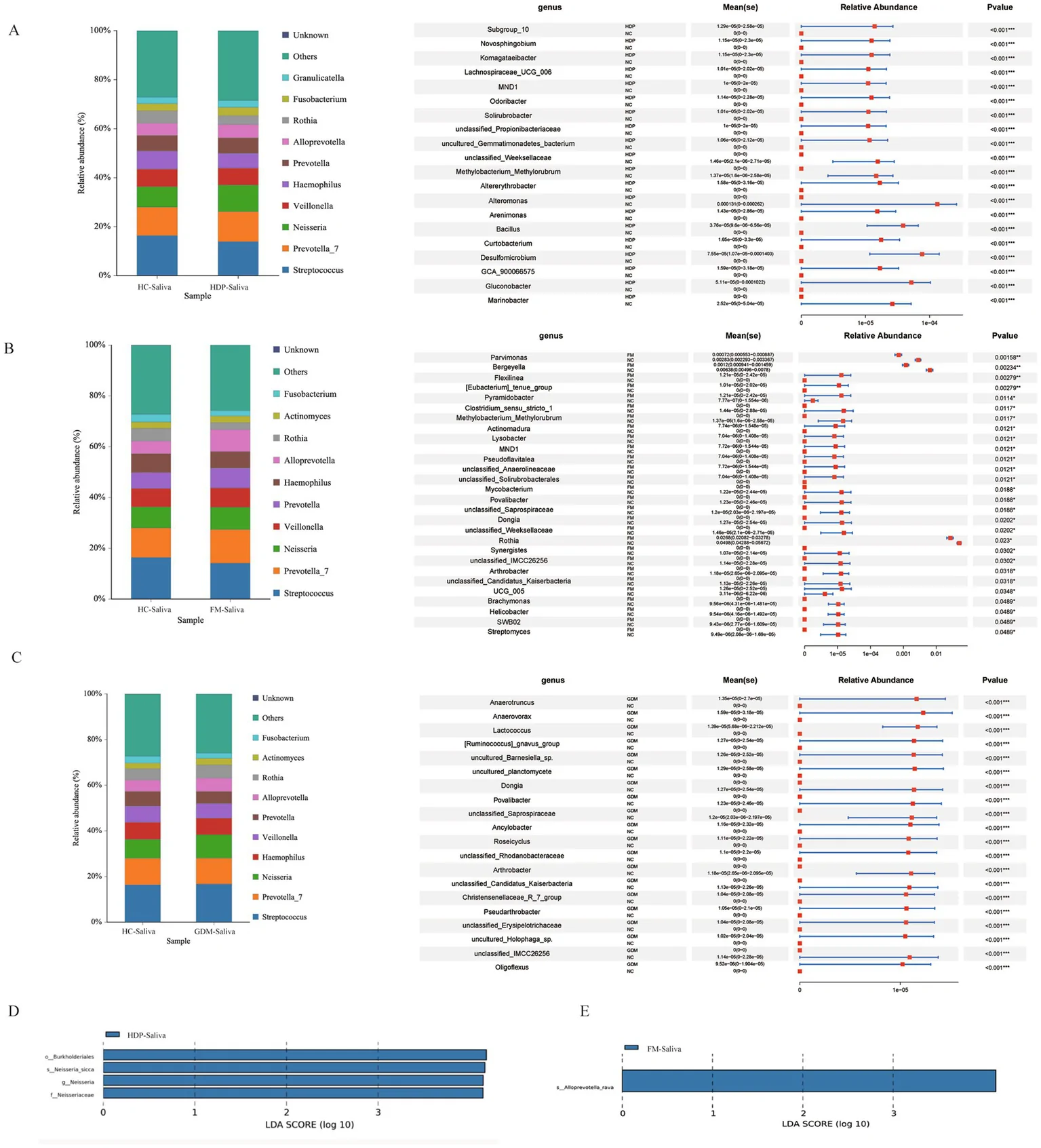
Comparison of salivary microbiota characteristics between patients with different pregnancy complications and NCs. **(A)** Bar plots showing the relative abundance of salivary microbiota at the genus level in the HDP group versus the NC group (left), and analysis of significantly different microbiota between the two groups (right). **(B)** Bar plots showing the relative abundance of salivary microbiota at the genus level in the FM group versus the NC group (left), and analysis of significantly different microbiota between the two groups (right). **(C)** Bar plots showing the relative abundance of salivary microbiota at the genus level in the GDM group versus the NC group (left), and analysis of significantly different microbiota between the two groups (right). **(D)** LEfSe analysis of differentially abundant microbiota between the HDP and the NC group (LDA score > 4). **(E)** LEfSe analysis of differentially abundant microbiota between the FM and the NC group (LDA score > 4).

### Differential cross-site concordance patterns along the saliva–amniotic fluid–meconium axis across pregnancy complications

To investigate whether different pregnancy complications affect the cross-site concordance of microorganisms among the maternal oral cavity, amniotic fluid, and neonatal gut, we analyzed the microbial composition, abundance changes, and shared taxa in saliva, amniotic fluid, and meconium across the HDP, FM, and GDM groups. The microbial community structures of the three sample types differed among the three complication groups. Unlike healthy individuals, saliva samples were clearly separated from amniotic fluid and meconium samples in all three complication groups, while amniotic fluid and meconium samples exhibited partial overlap (Figure 5A), suggesting a closer association between the intrauterine environment and the neonatal gut microbiota under disease conditions.

**Figure 5:**
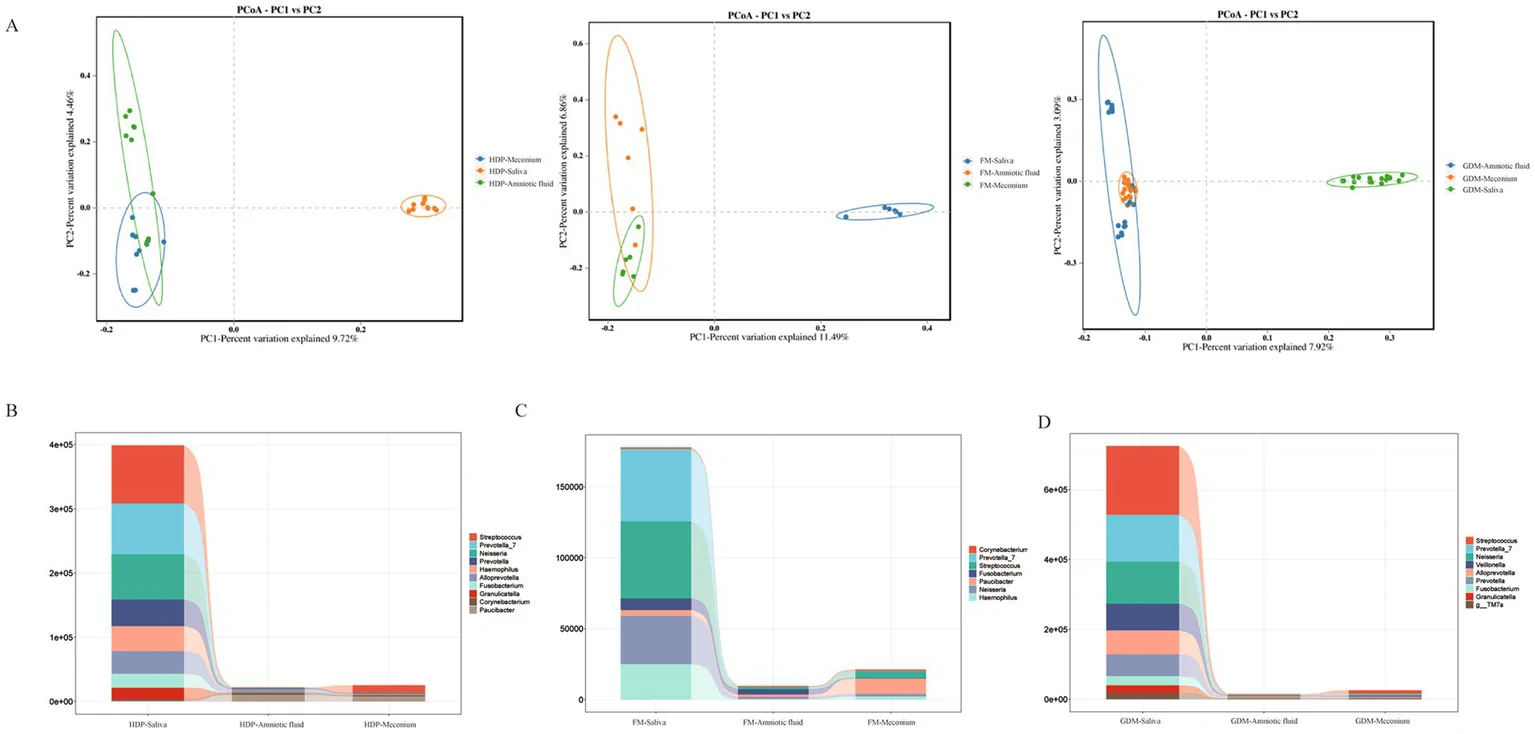
Cross-site microbial concordance along the saliva–amniotic fluid–meconium axis in patients with different pregnancy complications. **(A)** PCoA analysis of microbial communities in saliva, amniotic fluid, and meconium samples from different complication groups (HDP, FM, GDM).**(B–D)** Analysis of shared genus-level microbiota and their abundance change trajectories among saliva, amniotic fluid, and meconium in the HDP group **(B)**, FM group **(C)**, and GDM group **(D)**.

In the HDP group, a total of 142 shared microbial taxa were identified at the genus level among saliva, amniotic fluid, and meconium (Supplementary Figure S1A). Flow analysis of the top 10 shared taxa in terms of abundance changes revealed that *Prevotella*, *Neisseria*, *Haemophilus*, and *Streptococcus* exhibited distinct cross-site abundance patterns across saliva, amniotic fluid, and meconium (Figure 5B). These genera showed the highest abundance in saliva, maintained moderate abundance in amniotic fluid, and remained at relatively high levels in meconium.

In the FM group, 62 shared microbial taxa were identified (Supplementary Figure S1B). Flow analysis revealed that *Prevotella_7*, *Streptococcus*, *Fusobacterium*, *Paucibacter*, *Neisseria*, *Haemophilus*, and *Corynebacterium* exhibited distinct abundance changes across saliva, amniotic fluid, and meconium (Figure 5C). Among these, *Prevotella_7* and *Streptococcus* showed the highest abundance in saliva, maintained moderate abundance in amniotic fluid, and remained at relatively high levels in meconium. Fusobacterium maintained moderate abundance in amniotic fluid and showed higher abundance in meconium. Paucibacter showed low abundance in amniotic fluid but was significantly enriched in meconium. In contrast, *Neisseria*, *Haemophilus*, and *Corynebacterium* exhibited low abundances in both amniotic fluid and meconium.

In the GDM group, 217 shared microbial taxa were identified among saliva, amniotic fluid, and meconium (Supplementary Figure S1C). GDM-associated *Streptococcus*, *Prevotella*, *Fusobacterium*, and *Veillonella* exhibited distinct abundance changes across the three sites (Figure 5D). Among these, *Streptococcus*, *Prevotella*, and *Fusobacterium* showed the highest abundance in saliva, maintained moderate abundance in amniotic fluid, and remained at relatively high levels in meconium. In contrast, Veillonella showed low abundance in amniotic fluid but significantly rebounded in meconium.

### Multi-site candidate microbial signatures in different complications and their clinical relevance

To identify candidate microbial signatures for early warning of HDP and GDM and risk assessment of FM, LEfSe analysis (LDA score > 4.0) was performed to compare significantly enriched microbial taxa in saliva, amniotic fluid, and meconium among the NC, HDP, GDM, and FM groups. In *saliva*, *Rothia mucilaginosa* was significantly enriched in the HDP group, while *Neisseria sicca* was enriched in the GDM group (Figure 6A). In amniotic fluid, the HDP group showed significant enrichment of *Acinetobacter* and *Lactobacillus*, and the FM group showed enrichment of *Delftia* and *Parasutterella* (Figure 6B). In meconium, the FM group was enriched in *Comamonadaceae* and *Methyloversatilis,* the GDM group in *Acidobacteriota*, *Chloroflexi*, and *Acidobacteriae*, and the HDP group in *Enterobacter hormaechei*, *Gammaproteobacteria*, and *Enterobacterales* (Figure 6C).

**Figure 6:**
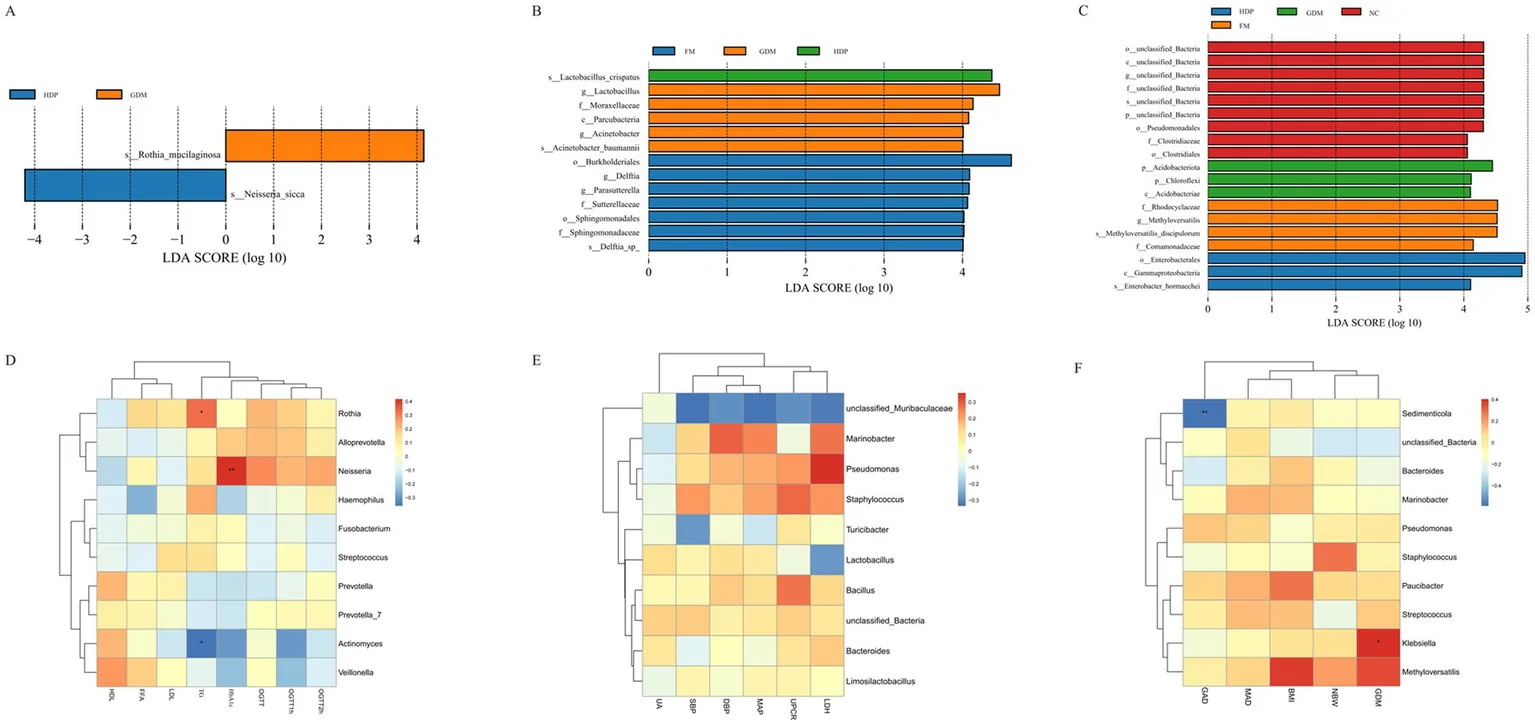
Clinical relevance of Multi-site candidate microbial signatures in different pregnancy complications. **(A–C)** Differential microbial analysis (LEfSe, LDA score > 4.0) in saliva **(A)**, amniotic fluid **(B)**, and meconium **(C)** samples between NCs and the HDP, GDM, and FM groups.**(D–F)** Correlation analysis between differential microbiota and clinical indicators in saliva **(D)**, amniotic fluid **(E)**, and meconium **(F)**.

To further evaluate the clinical value of candidate microbial signatures correlation analyses with key clinical indicators were performed. In saliva, *Rothia* was positively correlated with triglycerides, *Neisseria* with glycated hemoglobin, and Actinomyces was negatively correlated with triglycerides (*p* < 0.05) (Figure 6D). In amniotic fluid, Pseudomonas abundance was positively correlated with lactate dehydrogenase levels, *Marinobacter* with diastolic blood pressure, and *Bacillus* with urinary protein levels (*p* < 0.05) (Figure 6E). In meconium, *Methyloversatilis* was positively correlated with pre-pregnancy BMI, *Klebsiella* with diabetic status, *Staphylococcus* with neonatal birth weight, while *Sedimenticola* was negatively correlated with gestational age at delivery (*p* < 0.05) (Figure 6F).

## Discussion

Maternal–infant microbial transfer is increasingly recognized as a key determinant of neonatal gut microbiota establishment and early development. The maternal microbiota is regarded as the principal source of infant gut microbes, with transfer occurring during pregnancy and after delivery via multiple routes, including the birth canal, breast milk, and potentially intrauterine pathways (18). These routes facilitate maternal microbial colonization of the infant gut and shape early immune and metabolic programming (19). Nevertheless, whether pregnancy complications are associated with distinct cross-site microbial patterns remains poorly defined. In this study, we integrated paired maternal saliva, amniotic fluid, and neonatal meconium samples and characterized complication-specific microbial signatures and dynamic cross-site concordance patterns along the saliva–amniotic fluid–meconium axis.

A key question in neonatal microbiota development is whether the intrauterine environment is sterile and when initial microbial colonization occurs—during pregnancy or after birth. Although some studies suggest that fetal meconium lacks a detectable microbiota before birth (20), the presence of a microbiome in neonatal meconium remains debated (21). To ensure accurate detection and minimize contamination, we followed stringent protocols recommended for low-biomass samples, including sterile collection and rigorous data analysis procedures. In our NC group, the microbial composition of meconium was significantly distinct from that of saliva and amniotic fluid, while saliva and amniotic fluid exhibited relatively similar community structures. The dominance of Firmicutes and Proteobacteria in meconium, along with its higher alpha diversity compared to saliva and amniotic fluid, suggests that the establishment of the neonatal gut microbiota is a multi-source process. Notably, the compositional similarity between saliva and amniotic fluid at the phylum level—both rich in Actinobacteria, Bacteroidota, and Firmicutes—implies a possible migration of maternal oral microbiota to the amniotic fluid environment. Previous studies have isolated identical bacterial strains from the oral cavity of women with chorioamnionitis and their amniotic fluid, demonstrating that oral microorganisms may translocate to the uterus under specific pathological conditions (22). At the genus level, *Fusobacterium*, *Haemophilus*, *Prevotella_7,* and *Solobacterium* exhibited significantly higher relative abundances in saliva samples compared to amniotic fluid and meconium, consistent with the understanding that the oral cavity serves as the primary habitat for these genera. Their low abundances in amniotic fluid and meconium suggest that, even if potential cross-site transfer occurs, not all oral microorganisms are consistently detected in the intrauterine environment or the neonatal gut. Therefore, our findings support the view that neonatal meconium reflects microbial communities acquired during and after birth, while also suggesting a degree of microbial similarity between the maternal oral cavity and the intrauterine environment.

Another important finding of this study is that different pregnancy complications exert distinct effects on the neonatal meconium microbiome. We found that meconium microbial richness was significantly reduced in the HDP and FM groups, while the GDM group showed a decreasing trend that did not reach statistical significance. The HDP-associated reduction in alpha diversity may reflect the systemic impact of maternal hypertension on the intrauterine environment, thereby interfering with the initial colonization of the neonatal gut microbiota. At the genus level, the abundances of *Klebsiella*, *Enterobacter*, and *Sphingomonas* were significantly increased in the meconium of the HDP group. These genera are opportunistic pathogens, and their enrichment may suggest HDP-associated impairment of intestinal barrier function or immune dysregulation (23). A recent study on HDP also found increased abundances of *Enterobacter*, *Klebsiella*, and *Sphingomonas* in both maternal and neonatal microbiota of the HDP group, which is highly consistent with our meconium findings (24). Similarly, Chen et al. reported that *Escherichia*, Entero*c*occus, and *Klebsiella* were more prevalent in the gut microbiota of preterm infants with preeclampsia at 2 weeks of age (25). In the FM group, the relative abundances of *Staphylococcus* and *Methyloversatilis* were increased, and Prevotellaceae was significantly elevated. Xia et al. found that neonates in the GDM with FM group exhibited reduced Firmicutes and increased *Proteobacteria*, with lower Shannon index and OTUs compared to the normal birth weight group (26). Notably, the enrichment of *Methyloversatilis* in the FM group was consistent with its positive correlation with pre-pregnancy BMI in subsequent analyses, further supporting its potential as a candidate risk signature for macrosomia.

In the GDM group, the relative abundances of *Bifidobacterium* and *Staphylococcus* were higher than those in the NC group, while the abundance of Pseudomonas was lower. Liu et al. demonstrated that GDM mothers and their neonates exhibit unique microbial and metabolic characteristics, with 14 genera showing similar trends across both groups (27). Furthermore, *Bergeyella*, *Helicobacter*, *Mycobacterium*, and *Streptomyces* were significantly increased in the GDM group. These results support the notion that GDM-associated metabolic disturbances can exert early effects on the fetal gut microecology.

The composition of the amniotic fluid microbiome is considered a direct reflection of fetal intrauterine exposure. In this study, different pregnancy complications were each associated with specific alterations in the amniotic fluid microbiome. Emticicia was significantly increased in the HDP group, while Erysipelatoclostridiaceae was significantly elevated in the FM group, with LEfSe analysis further identifying Comamonadaceae as a differential taxon for the FM group. The GDM group showed enrichment of *Acinetobacter* and *Lactobacillus iners*. These specific alterations in the amniotic fluid microbiota may participate in the formation of an intrauterine inflammatory environment. Previous studies have shown that microbial invasion of the amniotic cavity and the resulting inflammation are important drivers of preterm birth (28). Our analysis suggests that different pregnancy complications may affect the intrauterine environment through distinct microbial mechanisms: HDP may be more associated with the enrichment of specific opportunistic pathogens, while GDM is linked to an abnormal increase in Lactobacillus species. The enrichment of *Lactobacillus iners* in the amniotic fluid of the GDM group is particularly noteworthy, as this species is closely associated with vaginal dysbiosis, and its presence in amniotic fluid may indicate an increased risk of ascending infection (29). The oral microbiome, serving as a window into systemic metabolic and inflammatory status, showed specific associations with different complications in this study. The microbial composition of saliva samples was similar across all groups, predominantly consisting of *Veillonella*, *Neisseria*, *Prevotella*_*7*, *Haemophilus*, *Alloprevotella*, and *Streptococcus*. However, differential analysis revealed complication-specific alterations. In the HDP group, *Desulfomicrobium*, *Novosphingobium*, and *Lachnospiraceae* were significantly increased, and LEfSe analysis further confirmed the enrichment of *Neisseria* and *Neisseria sicca*. Du Toit et al. found that patients with preeclampsia have lower oral nitrate-reducing bacterial activity, reduced salivary nitrite levels, and significantly lower plasma nitrate levels compared to controls (30). Similarly, Azevedo et al. reported a decreased abundance of nitrate-reducing microorganisms in the oral cavity of hypertensive pregnant women (31). Our findings are consistent with these reports, suggesting that oral dysbiosis may contribute to blood pressure regulation by affecting nitric oxide bioavailability. In the FM group, *Streptococcus*, *Rothia*, and *Parvimonas* were significantly increased in saliva, and LEfSe analysis identified *Alloprevotella_ravazai* as significantly elevated. Notably, *Rothia* showed consistent changes across multiple sites (saliva, amniotic fluid, and meconium) in the FM group, suggesting its potential as a cross-site candidate microbial signature in FM. In the GDM group, *Lactococcus* and *Arthrobacter* were significantly increased in saliva. The gut microbiome of GDM patients exhibits distinct compositional features, with specific bacterial species such as *Bacteroides dorei* positively correlated with glucose levels at multiple time points during the OGTT (32). Our salivary findings suggest that the oral microbiota may also serve as a potential source of candidate microbial signatures for GDM.

The most significant finding of this study is the revelation of distinct cross-site concordance patterns along the saliva–amniotic fluid–meconium axis under different complication statuses. In disease states, saliva samples were clearly separated from amniotic fluid and meconium samples, while amniotic fluid and meconium exhibited partial overlap, suggesting that disease conditions may enhance the association between the intrauterine environment and the neonatal gut microbiota. Previous studies have elucidated the “maternal gut-breast milk-infant gut” microbial transmission pathway, demonstrating that *bifidobacterium* is a key genus in maternal–infant vertical transmission (33). In the HDP group, 142 shared microbial taxa were identified among saliva, amniotic fluid, and meconium. *Prevotella*, *Neisseria*, *Haemophilus*, and *Streptococcus* exhibited a continuous abundance gradient across saliva, amniotic fluid, and meconium, with the highest abundance in saliva, moderate abundance in amniotic fluid, and relatively high abundance in meconium. Yang et al. similarly found parallel changes in maternal and neonatal microbiota in the HDP group, indicating HDP-associated intergenerational concordance (24). In the FM group, 62 shared microbial taxa were identified. *Prevotella_7, Haemophilus*, and *Streptococcus* also exhibited continuous abundance gradient, while *Fusobacterium* maintained moderate abundance in amniotic fluid and further increased in meconium, suggesting a pattern potentially consistent with intrauterine detection and subsequent presence in the neonatal gut. Paucibacter showed low abundance in amniotic fluid but was significantly enriched in meconium, suggesting that it may be associated with maternal contact during delivery rather than prenatal cross-site transfer. In the GDM group, as many as 217 shared microbial taxa were identified among saliva, amniotic fluid, and meconium—far exceeding the numbers in the HDP and FM groups. This results suggests that GDM may be associated with more extensive cross-site microbial concordance. Low et al. demonstrated intergenerational transfer of GDM-associated gut dysbiosis from mothers to infants (34). *Streptococcus*, *Prevotella*, and *Fusobacterium* exhibited a continuous abundance gradient across saliva, amniotic fluid, and meconium, whereas Veillonella showed low abundance in amniotic fluid but a marked increase in meconium, indicating a distinct cross-site abundance pattern.

Furthermore, we identified complication-specific candidate microbial signatures across multiple sites. Importantly, the correlations of these candidate microbial signatures with clinical indicators enhanced their clinical value. In saliva, Rothia was positively correlated with triglycerides, *Neisseria* with glycated hemoglobin, while *Actinomyces* was negatively correlated with triglycerides. Studies have demonstrated that specific taxa in the gut microbiota of women with gestational diabetes mellitus are significantly associated with oral glucose tolerance test results (35). In amniotic fluid, *Pseudomonas* abundance was positively correlated with lactate dehydrogenase, *Marinobacter* with diastolic blood pressure, and *Bacillus* with urinary protein levels. Chen et al. reported significantly reduced gut microbial diversity and increased abundance of specific opportunistic pathogenic genera in women with preeclampsia (36), and our correlation analysis from the perspective of the amniotic fluid microbiome supports this view. In meconium, *Methyloversatilis* was positively correlated with pre-pregnancy BMI, *Klebsiella* with diabetic status, and *Staphylococcus* with neonatal birth weight, while Sedimenticola was negatively correlated with gestational age at delivery. Zheng et al. demonstrated that several genera and metabolites in GDM mothers and their neonates show significant correlations with clinical phenotypes (37). These correlation analyses support, from a microbiological perspective, the association of different complications with specific metabolic and inflammatory pathways. In this study, the differential genera associated with different pregnancy complications showed little overlap across sites, with HDP, FM, and GDM groups each exhibiting distinct specific genera in meconium, amniotic fluid, and saliva. This phenomenon suggests that the effects of different complications on the maternal and neonatal microbiome are site-specific, potentially reflecting the unique pathophysiological mechanisms of each ecological niche. However, *Staphylococcus* and *Streptococcus* showed increased trends across multiple sites (meconium, amniotic fluid, and saliva) in the FM group, suggesting that these genera may have cross-site associations with excessive fetal growth, warranting further investigation.

This study has several limitations. First, the relatively small sample size, particularly the inclusion of only 6 cases in the FM group, may limit statistical power and the reliability of subgroup analyses. Second, dietary and nutritional data were not systematically collected, and other clinical characteristics were not incorporated as covariates; their potential contribution cannot be fully excluded. Future studies with larger sample sizes should re-evaluate these findings using multivariable models. Third, the taxonomic resolution of 16S rRNA gene sequencing is limited to the genus level and cannot distinguish strain-level transfer or colonization. In addition, extraction blanks were not systematically included, and because amniotic fluid is a low-biomass sample, total bacterial load was not quantified independently of 16S rRNA amplicon sequencing, which may affect the interpretation of the amniotic fluid microbiome. Future studies with larger sample sizes, longitudinal designs, strain-resolved metagenomic sequencing, absolute quantification methods, and additional contamination controls are needed to validate these findings and further clarify the potential mechanisms underlying cross-site microbial patterns.

## Conclusion

In summary, this study provides the first systematic comparison of the associations between three major pregnancy complications (GDM, HDP, and FM) and the microbiota of maternal saliva, amniotic fluid, and neonatal meconium, revealing complication-specific microbial signatures across different sites and delineating the dynamic cross-site concordance patterns along the saliva–amniotic fluid–meconium axis in disease states. Our findings offer new insights into the microbiological patterns associated with pregnancy complications and lay a foundation for developing microbiota-based early warning strategies.

## Data Availability

The sequencing data generated in this study have been deposited in the NCBI Sequence Read Archive (SRA) under BioProject accession number PRJNA1542473.

## Footnotes

1
https://www.biocloud.net

## Supplementary material

SUPPLEMENTARY FIGURE S1 Shared genus-level microbiota among saliva, amniotic fluid, and meconium in different complication groups. **(A–C)** Venn diagrams showing shared genus-level microbiota in saliva, amniotic fluid, and meconium samples from the HDP group **(A)**, FM group **(B)**, and GDM group **(C)**.

SUPPLEMENTARY TABLE S1 Baseline clinical characteristics of the NC, GDM, FM, and HDP groups.
