# Supplementary material for: Complication-specific microbial signatures in gestational diabetes mellitus, hypertensive disorders of pregnancy, and fetal macrosomia: a multi-site analysis of the maternal saliva–amniotic fluid–neonatal meconium continuum
Source: Front Nutr. 2026 Oct 21;13:1961007. doi: 10.3389/fnut.2026.1961007 (PMC13731367; doi:10.3389/fnut.2026.1961007)
Supplement: Supplementary file 1 [file Data_Sheet_1.PDF]

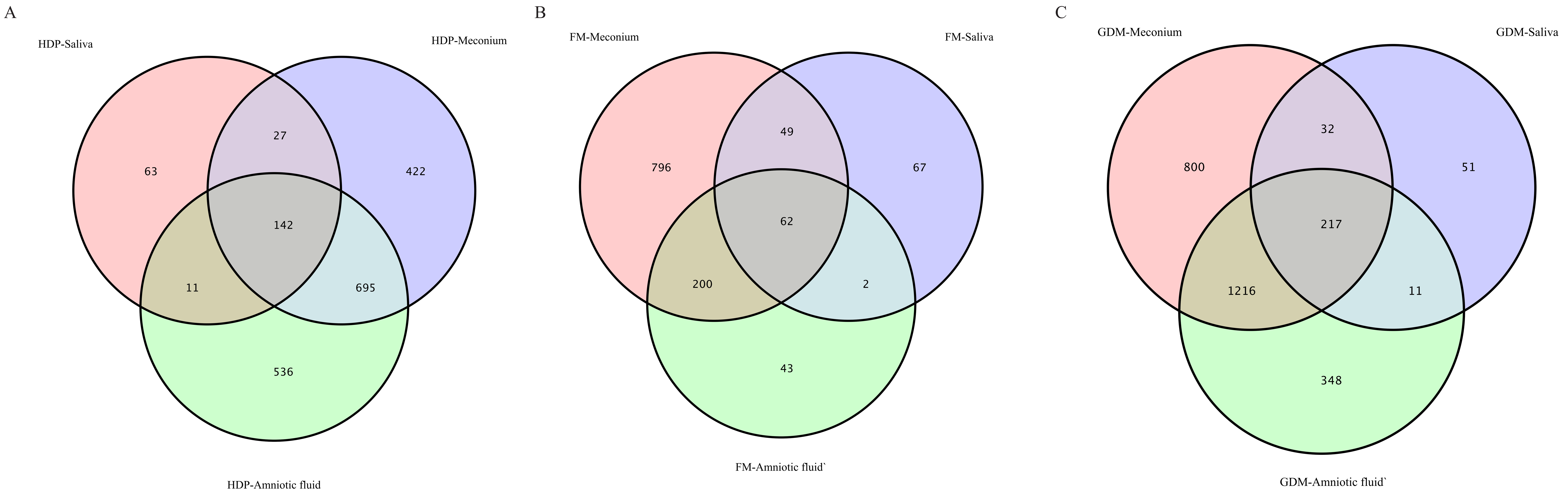

A
HDP-Saliva
HDP-Meconium
63
27
422
142
11
695
536
HDP-Amniotic fluid
B
FM-Meconium
FM-Saliva
796
49
67
62
200
2
43
FM-Amniotic fluid`
C
GDM-Meconium
GDM-Saliva
800
32
51
217
1216
11
348
GDM-Amniotic fluid`
